# Polyphenols from the Mediterranean herb rosemary (*Rosmarinus officinalis*) for prostate cancer

**DOI:** 10.3389/fphar.2013.00029

**Published:** 2013-03-25

**Authors:** Sakina M. Petiwala, Angela G. Puthenveetil, Jeremy J. Johnson

**Affiliations:** ^1^Department of Pharmacy Practice, College of Pharmacy, University of Illinois at ChicagoChicago, IL, USA; ^2^University of Illinois Cancer CenterChicago, IL, USA

**Keywords:** carnosol, carnosic acid, prostate cancer, rosemary extract, diterpene

## Abstract

The Mediterranean diet is rich in fruits and vegetables and has been associated with a variety of health benefits including cancer prevention. One aspect of the diet that has not received enough attention is Mediterranean herbs. Specifically, rosemary and its polyphenolic diterpenes (carnosic acid and carnosol) are known to possess anti-oxidant activity that may be beneficial for cancer control. Herein, we describe the *in vitro* and *in vivo* studies carried out towards understanding the molecular mechanisms of carnosic acid and carnosol leading to inhibition of prostate cancer. The reported findings suggest that these polyphenols target multiple signaling pathways involved in cell cycle modulation and apoptosis. Further work is required to understand its potential for health promotion and potential drug discovery for prostate cancer chemoprevention.

## MEDITERRANEAN DIET AND PROSTATE CANCER

The Mediterranean diet has received significant attention for its metabolic and cardiovascular health promoting benefits (Piscopo, 2009). The traditional Mediterranean diet primarily consists of a high intake of fresh fruits and vegetables, legumes, non-refined cereals including bread, fish, olive oil, a moderate consumption of red wine, and a low consumption of red meats (Keys et al., 1986). There have been multiple epidemiological studies evaluating various components of this diet. However, an area that has not received enough attention is the use of culinary herbs including rosemary, basil, oregano, sage, and others. One herb in particular, rosemary (*Rosmarinus officinalis*), has long been associated with anti-inflammatory and anti-oxidant properties. A population-based study has even suggested that an overall risk reduction in cancer incidence is correlated with patients consuming herbs including rosemary (OR = 0.66, 95% CI = 0.37–1.15; Fortes et al., 2003).

Recently, the European Union (EU) has approved the use of rosemary extracts for food preservation and has been adopted into the EU food additive legislation (Aguilar et al., 2008). The study was done using rosemary extracts standardized to carnosic acid and carnosol, which have been noted for their strong anti-oxidant activity. Multiple extracts have been evaluated for their acute and chronic toxicity suggesting they are very tolerable. Acute oral toxicity testing of a rosemary extract at doses as high as 8.5 and 10.5 g/kg in male and female rats, respectively, did not induce mortality. In a 13-week oral study in male and female rats the NOAEL (no observable adverse event level) of different rosemary extracts was between 180 and 400 mg/kg body weight/day, which was equivalent to 20–60 mg/kg/day of carnosol and carnosic acid per day. The adult mean intake was estimated to be between 500 and 1,500 mg of carnosol and carnosic acid per day. The panel concluded that the use of rosemary as a preservative/additive was not a safety concern. In the United States, rosemary has also been categorized by the FDA as “generally recognized as safe” or GRAS (CFR182.10; 182.20).

Rosemary itself contains a variety of polyphenolic compounds including carnosol, carnosic acid, methyl carnosol, rosmarinic acid, ursolic acid, and many others (Lo et al., 2002). Approximately 5% of the dry weight of rosemary leaves is comprised of the phenolic diterpenes carnosol and carnosic acid (Huang et al., 1994). They are estimated to account for >90% of the anti-oxidant activity (Aruoma et al., 1992). Carnosic acid, which is the most abundant diterpene, has been shown to be well absorbed orally in rats (90 mg/kg) with a maximum serum concentration of 126 μM and an absolute oral bioavailability of 65.1% (Piscopo, 2009). The anti-oxidant properties are seen through its ability to chelate iron, scavenge peroxyl free radicals and inhibit lipid peroxidation (Đilas et al., 2012). Carnosol is a derivative of carnosic acid containing a lactone ring. It has been known to have anti-inflammatory, anti-oxidant, anti-microbial, and anti-cancer properties. The other phytochemicals are also known to possess similar properties. For example, rosmarinic acid has been known to have strong radical scavenging effect (Aruoma et al., 1992). Ursolic acid is a triterpenoid compound that has been shown to have anti-inflammatory and even an anti-depressant effect (Machado et al., 2012). Carnosol exhibits anti-inflammatory properties through its ability to reduce leukotrienes, inhibit 5-lipoxygenase, antagonize the intracellular Ca^2^^+^ mobilization, and inhibit the secretion of leukocyte elastase (Poeckel et al., 2008). In traditional Chinese medicine, rosemary extracts which contain a high amount of diterpenes and triterpenes have been used to treat inflammatory conditions. Additionally, a variety of dietary supplements of rosemary extracts are available that have been standardized to carnosol and/or carnosic acid. The clinical implications of these phytochemicals are far-reaching and still undergoing analysis including the treatment of different cancers.

Prostate cancer is an example of a potential use of rosemary for chemoprevention and tumor reduction. It is a good candidate for chemoprevention because it is typically diagnosed in men over the age of 50 suggesting that even a minimal delay in prostate cancer development through pharmacological or nutritional manipulation could greatly reduce the incidence of clinically detectable disease (Johnson et al., 2008). Prostate cancer has a long latency period and a tendency to metastasize via the blood stream (Joshua et al., 2008; Schroder et al., 2009; Siegel et al., 2011). Autopsy studies have shown that many young men in their 20s and 30s have developed pre-cancerous lesions (Yatani et al., 1982). Prostate cancer is a proliferation of epithelial cells which are seen to have a chromosomal gain or loss mutation, leading to uncontrolled cell growth. It is a slow growing cancer that follows the same progression as many epithelial cancers from mild dysplasia to invasion of the basement membrane (Umar et al., 2012). There are three classifications with the first form being latent and benign, the second form being moderately progressive and the third form being rapidly progressive and extremely malignant (Waterbor and Bueschen, 1995). The latest statistics among males in the USA estimates about 240,890 new cancer cases and 33,720 mortalities due to prostate cancer alone in 2011 (Siegel et al., 2011).

## ANTI-CANCER ACTIVITY OF CARNOSIC ACID TOWARDS PROSTATE CANCER

Carnosic acid, a natural diterpene, alone constitutes 1.5–2.5% of dried leaves of rosemary. Well-known as one of the most potent anti-oxidant agents of rosemary, carnosic acid also exhibits effective anti-cancer properties. It has significant growth inhibitory and cytotoxic properties in prostate cancer cell lines, DU-145 and PC3, decreasing the cell viability of the cell lines to 13 and 20%, respectively, when treated for 48 h at a concentration of 6.25 μg/ml (i.e., 18.8 μM; Yesil-Celiktas et al., 2010).

Another study by Kar et al. (2012) also illustrates the anti-proliferative and cytotoxic properties of carnosic acid in a concentration and time-dependent manner in DU-145 and PC3 prostate cancer cell lines as determined by MTT (3-(4,5-dimethylthiazol-2-yl)-2,5-diphenyltetrazolium bromide, a yellow tetrazole) assay. At a concentration of 80 μM (i.e., 26.6 μg/ml), carnosic acid resulted in >95 and 90% cell death in PC3 and DU-145 cells, respectively. The half maximal inhibitory concentration (IC_50_) value for PC3 cells was found to be 41.1 μM. Carnosic acid induced apoptosis as evidenced by DNA fragmentation, terminal deoxynucleotidyl transferase dUTP nick end labeling (TUNEL) staining and Annexin V–PI flow cytometric analysis. At 80 μM, carnosic acid promoted apoptosis in PC3 cells with 56.1% apoptosis compared to 47.2% apoptosis in DU-145 cells (Kar et al., 2012). Carnosic acid also increased expression of the pro-apoptotic protein Bax and decreased the anti-apoptotic Bcl-2 protein leading to the release of cytochrome *c* from the mitochondria suggestive of a mitochondrial-dependent apoptosis. The modulation of caspases by carnosic acid appears to be cell-type dependent. Carnosic acid induces apoptosis through both pathways in PC3 cells as seen by the upregulation of caspases-8, -9, and -3 by 8.2-, 5.1-, and 10.1-fold after 36 h of treatment. Although expression of caspase-9 increased by 9.3-fold in DU-145 cells, no change in expression of caspase-8 was observed in these cells suggesting activation of only intrinsic mediated apoptotic pathway in androgen refractory DU-145 cell line. Furthermore, carnosic acid was also found to trigger apoptosis via inhibition of phosphatidylinositide 3-kinase (PI3K)/Akt signaling pathway which in turn suppresses IκB kinase/nuclear factor kappa B (IKK/NF-κB) pathway in PC3 prostate cancer cells (Kar et al., 2012).

Besides prostate cancer, carnosic acid is also cytotoxic against various cancer cell lines derived from human leukemia, breast, lung, and liver malignant tissues (Yesil-Celiktas et al., 2010). Some *in vitro* studies have shown the anti-cancer activities of carnosic acid against human neuroblastoma IMR-32 cells (Tsai et al., 2011), colonic adenocarcinoma Caco-2 cells (Visanji et al., 2006), and myeloid leukemia HL-60 cells (Steiner et al., 2001). A possible *in vivo* chemopreventive effect of carnosic acid has been described in golden Syrian hamsters against 7,12-dimethylbenz(a)anthracene (DMBA)-induced oral carcinogenesis (Manoharan et al., 2010). At an oral dose of 10 mg/kg body weight/day, carnosic acid almost completely prevented formation of oral carcinoma in hamster’s buccal mucosa compared to 100% tumor formation in control animals. Levels of phase I and phase II detoxification enzymes were found to increase and decrease in control hamsters, respectively, whereas levels of these biomarkers were restored to normal ranges in carnosic acid treated animals. Also, the level of anti-oxidant enzymes were reduced in control group compared to the treated group. These results suggest that inhibition of DMBA-induced oral cancer might be due to anti-oxidant effect and removal of the toxic metabolite of DMBA by carnosic acid (Manoharan et al., 2010).

## ANTI-CANCER ACTIVITY OF CARNOSOL TOWARDS PROSTATE CANCER

Carnosol is an *ortho*-diphenolic diterpene with an abietane carbon skeleton that is the product of oxidative degradation of carnosic acid (Gajhede et al., 1990; Johnson, 2011). Together, carnosic acid and carnosol are responsible for nearly all of the anti-oxidant activity of rosemary. Carnosol has been reported to possess numerous pharmacological properties including anti-inflammatory, anti-oxidant, and anti-tumor activities.

However, the anti-tumorigenic effects of carnosol on prostate cancer have only recently been explored. Work in our laboratory has demonstrated the anti-proliferative properties of carnosol in a dose and time-dependent manner on PC3 cells with an observed IC_50_ value of 48.3, 39.2, and 34 μmol/l at 24, 48, and 72 h, respectively. Carnosol led to an induction of cell cycle arrest at G_2_ phase of the cell cycle in PC3 cells along with increase in expression levels of cell cycle regulatory proteins, p21 and p27, and a simultaneous decrease in protein levels of cyclin-A, -D1, and -D2 and cyclin-dependent kinase (cdk) proteins-2 and -6 (Johnson et al., 2008).

Our study further revealed how carnosol modulates multiple signaling pathways in a single cell line. Concomitant with cell cycle arrest, we also observed upregulation of Bax and downregulation of Bcl-2 and pro-caspase 8 suggesting occurrence of apoptosis upon treatment of carnosol in PC3 cells. Besides activating the intrinsic apoptotic pathway, carnosol also inhibited the PI3K/Akt pathway. At a dose of 20 and 40 μM, carnosol showed a dramatic decrease in the protein expression of Akt at phosphorylation sites Thr-308 and Ser-473, respectively. In addition to PI3K/Akt inhibition, carnosol also activated the 5^′^ adenosine monophosphate-activated protein kinase (AMPK) pathway. The expression of AMPK beta-1 regulatory subunit was found to increase by 365% in carnosol treated cells compared to control cells via a protein array. AMPK and its downstream molecule mammalian target of rapamycin (mTOR) have been considered as a therapeutic target for cancer treatments. Typically mTOR protein is upregulated in prostate cancer. Upon treatment of PC3 cells with carnosol we observed a dose-dependent decrease in phosphorylation of mTOR protein thereby leading to inhibition of prostate cancer *in vitro* (Johnson et al., 2008).

Additionally, our *in vivo* studies have demonstrated that carnosol when given orally at a dose of 30 mg/kg inhibits the growth of prostate cancer in athymic nude mice by 36% along with a 26% decrease in serum prostate-specific antigen (PSA) levels compared to untreated control animals (Johnson et al., 2010). This study also highlights a unique property of carnosol wherein it functions as a dual disruptor of both androgen receptor (AR) and estrogen receptor α (ERα) *in vitro* in prostate cancer cells (LNCaP and 22Rv1) as well as *in vivo* in nude mice (Johnson et al., 2010). Using a time-resolved fluorescence resonance energy transfer (TR-FRET) assay we found that carnosol can bind to both AR and ERα and displays antagonist activity at both the receptors without any agonistic properties associated with it. More than 35 agents have been evaluated as dual disruptors of AR and ERα, however, to the best of our knowledge, this is the first report of an agent that possesses solely antagonistic properties (Wilkinson et al., 2008).

## CONCLUSION

This review focuses on the Mediterranean herb, rosemary, its polyphenolic diterpenes (carnosic acid and carnosol) and their role in chemoprevention of prostate cancer. Epidemiological studies suggest a reduced risk of cancer in patients consuming rosemary. Herein, we have described the mechanism by which carnosic acid and carnosol inhibits prostate cancer. Essentially both diterpenes inhibit cancer by promoting apoptosis and inhibiting the critical PI3K/Akt signaling pathway which is an important regulator of tumor cell survival. These findings warrant further research to understand the potential of rosemary as a cancer chemopreventive agent in prostate cancer.

## Conflict of Interest Statement

The authors declare that the research was conducted in the absence of any commercial or financial relationships that could be construed as a potential conflict of interest.
